# Alcoholic Paralysis

**Published:** 1889-12-21

**Authors:** 


### ALCOHOLIC PARALYSIS.

"The Premonitory Symptoms of Alcoholic Paralysis" (*Lancet*) is the title of a communication from the pen of Dr. James Ross, physician to the Royal Infirmary, Manchester. The premonitory symptoms are, disorders of the "tactile sensibilities of the extremities," which patients usually describe as numbness of the fingers and toes, vaso-motor spasms of the extremities named by Raynaud "local asphyxia," and which the patients refer to as deadness and coldness of the fingers and toes; also severe cramps, most frequent and severe in the muscles of the calves. Such symptoms may generally be discovered long before the ordinary symptoms occur, such as bloated face, lightening pains, morning retching, loss of the patella, tendon reactions, etc. As Dr. Ross truly observes, "Nothing tends so much on the treatment of a disease as an early recognition of the symptoms of its inception," and it is, therefore, well to bear in mind that incipient alcoholic paralysis may often be recognised in a very early stage by the premonitory symptoms detailed above.

